# Remifentanil Attenuates LPS-Induced Genital Tract Injury by Modulating Inflammation, Oxidative Stress, and Mitochondrial Gene Expression in a Rat Sepsis Model

**DOI:** 10.1007/s43032-025-01930-7

**Published:** 2025-07-15

**Authors:** Ozlem Ozmen, Halil Asci, Senay Topsakal, Rumeysa Taner, Mert Oktem, Kadriye Nilay Ozcan, Bunyamin Aydin

**Affiliations:** 1 Department of Pathology, Faculty of Veterinary Medicine, Burdur Mehmet Akif Ersoy University, Burdur, Türkiye; 2 Department of Pharmacology, Faculty of Medicine, Suleyman Demirel University, Isparta, Türkiye; 3 Faculty of Medicine, Department of Endocrinology and Metabolism, Pamukkale University, Denizli, Türkiye; 4 Department of Bioengineering, Institute of Natural and Applied Sciences, Suleyman Demirel University, Isparta, Türkiye; 5 Department of Gynecology and Obstetrics, Faculty of Medicine, Suleyman Demirel University, Isparta, Türkiye; 6 Department of Internal Medicine, Kütahya University of Health Sciences, Kütahya, Türkiye

**Keywords:** Remifentanil, LPS, Oxidative Stress, Apoptosis, Inflammation, Caspase, NRF2, Genital Tract, Rat Model

## Abstract

Lipopolysaccharide (LPS), a key component of Gram-negative bacterial cell walls, triggers strong inflammatory responses that can severely impair reproductive organs. The female genital tract—including the ovaries, fallopian tubes, and uterus—is particularly vulnerable to LPS-induced inflammation, oxidative stress, and apoptosis. This study investigated the protective effects of remifentanil (REMI), an ultra-short-acting opioid with anti-inflammatory and antioxidant properties, against LPS-induced injury in female Wistar albino rats. Thirty-two rats were divided into four groups: LPS, LPS + REMI, REMI, and Control. LPS (5 mg/kg, i.p.) and REMI (0.04 mg/kg, i.v.) were administered, followed by histopathological, immunohistochemical (TNF-α, NF-κB, iNOS), and RT-qPCR analyses (Caspase-3, -9, -12, NRF2, HO-1) of the ovaries, uterus, and fallopian tubes. LPS exposure caused severe histological damage, increased inflammatory markers, and elevated caspase activity, particularly in the fallopian tubes and uterus. REMI treatment significantly reduced these pathological changes, restored NRF2 and HO-1 expression, and suppressed caspase activation. These findings indicate that REMI effectively protected against LPS-induced genital tract injury by modulating inflammation, reducing oxidative stress, and preventing apoptosis, highlighting its potential as a therapeutic agent for sepsis-related reproductive organ damage.

**Supplementary Information:**

The online version contains supplementary material available at 10.1007/s43032-025-01930-7.

## Introduction

Gram-negative bacterial cell walls contain lipopolysaccharide (LPS), a potent endotoxin that robustly activates the immune system. Upon recognition by Toll-like receptor 4 (TLR4) on immune cells, LPS initiates downstream signaling cascades involving the activation of nuclear factor kappa B (NF-κB), a key transcription factor regulating genes associated with inflammation and immune responses [1, 2]. Under resting conditions, NF-κB remains sequestered in the cytoplasm by its inhibitor IκB. However, LPS stimulation promotes IκB phosphorylation and degradation, enabling NF-κB to translocate into the nucleus and induce the transcription of pro-inflammatory genes, including tumor necrosis factor-alpha (TNF-α), interleukins, and adhesion molecules [3, 4]. This pro-inflammatory transcriptional program amplifies tissue inflammation and damage by promoting leukocyte infiltration, increasing vascular permeability, and enhancing cytokine release [5].

A critical downstream effector in LPS-induced signaling is inducible nitric oxide synthase (iNOS), transcriptionally regulated by NF-κB. iNOS catalyzes the sustained production of nitric oxide (NO), a reactive free radical involved in vasodilation and immune defense. However, excessive NO levels contribute to oxidative stress and cytotoxicity through the formation of peroxynitrite, which damages lipids, proteins, and DNA [6]. Thus, the NF-κB/iNOS axis represents a central pathway mediating LPS-induced inflammation and oxidative tissue injury [7].

The female reproductive system—particularly the ovaries, fallopian tubes, and uterus —is highly sensitive to such inflammatory insults. LPS exposure disrupts the structural and functional integrity of these organs, leading to cellular degeneration, edema, vascular congestion, and epithelial loss [8]. At the molecular level, these inflammatory conditions activate apoptotic pathways, notably those involving Caspase-3 (Cas-3), Caspase-9 (Cas-9), and Caspase-12 (Cas-12). Cas-9 is involved in the intrinsic (mitochondrial) pathway of apoptosis, while Cas-12 is associated with endoplasmic reticulum (ER) stress-induced apoptosis, both contributing to LPS- induced cell death [9, 10].

Oxidative stress also plays a central role in mediating LPS-induced damage. The nuclear factor erythroid 2–related factor 2 (NRF2)/heme oxygenase-1 (HO-1) pathway is a key regulator of cellular redox homeostasis. Under oxidative stress, NRF2 dissociates from its cytoplasmic inhibitor Keap1 and translocates to the nucleus, where it activates the transcription of antioxidant and cytoprotective genes, including HO-1. However, LPS has been shown to suppress the NRF2/HO-1 axis, there by weaking the antioxidant defense system and exacerbating tissue injury [11, 12]. This suppression increases the susceptibility of reproductive tissues to oxidative damage [13].

To counteract LPS-induced inflammation, agents with anti-inflammatory, antioxidant, and anti-apoptotic properties are under active investigation. Remifentanil (REMI), a short-acting opioid, has demonstrated not only analgesic effects but also significant anti-inflammatory and antioxidant activities [14–16]. Its anti-inflammatory effects are largely attributed to suppression of pro-inflammatory cytokine production via inhibition the NF-κB pathway [17]. Moreover, REMI has been reported to enhance NRF2 and HO-1 expression, bolstering the cell’s antioxidant defenses [18]. By modulating oxidative stress and inflammatory signaling, REMI may protect against apoptosis and mitochondrial dysfunction in reproductive tissues.

Although the protective effects of REMI have been demonstrated in organs such as the lungs, liver, and kidneys, its impact on the female reproductive tract have not been thoroughly explored [14–19]. This study aims to evaluate the protective effects of REMI against LPS-induced inflammation, oxidative stress, and apoptosis in the ovaries, fallopian tubes, and uterus using an established rat model of endotoxemia. To our knowledge, this is the first study to comprehensively assess REMI’s protective actions, the findings may provide novel therapeutic insights for the management of sepsis-related reproductive dysfunction.

## Materials and Methods

### Ethical Approval

All animal experiments in this study were conducted in accordance with the ARRIVE guidelines and EU Directive 2010/63/EU for animal research. The experimental protocol was approved by the Suleyman Demirel University Local Animal Experimentation Ethics Committee (Approval No: 06.03.2025/03-450). This study was funded by the Scientific Research Projects Coordination Unit at Suleyman Demirel University (Project Code: TSG-2024-9515).

### Animals and Experimental Procedure

A total of 32 female Wistar albino rats, aged 10 weeks, were used. Based on relevant parameters (α = 0.05, 1-β = 0.90, effect size = 0.32), a sample size of four groups with eight rats each was calculated using G*Power 3.1.9.7 software. The rats were housed in four standard Euro-type cages under controlled conditions: 12-hour light/dark cycle, temperature of 21–22 °C, and 55% relative humidity. All animals were provided with standard commercial chow (Korkuteli yem/Antalya, Türkiye).

### Study Design

The rats (*n* = 8 per group), obtained from the Suleyman Demirel University Experimental Animal Research Laboratory, were randomly assigned to four groups based on body weight. Randomization was applied during group allocation to minimize bias, and blinding was maintained during tissue processing and analysis.

Prior to the experiments, the estrous cycle phases of each rat was determined via daily macroscopic examination of the vagina and vaginal smear analysis. Rats in the diestrus phase were identified based on cytological criteria, specifically the predominance of leukocytes and the absence of cornified or nucleated epithelial cells [20].

*Control (CONT) group* (*n* = 8): Rats received an intraperitoneal (i.p.) injection of 0.5–1 ml of saline. Four hours later, they were given a 2 ml intravenous (IV) infusion of saline over 40 min. Euthanasia was performed six hours after the initial saline administration.

*LPS group* (*n* = 8): Rats received an i.p. injection of 5 mg/kg LPS dissolved in saline [14]. Four hours later, they received a 2 ml IV infusion of saline over 40 min. Euthanasia was performed six hours after the LPS administrations.

*LPS-REMI group* (*n* = 8): Rats received an i.p. injection of 5 mg/kg LPS. Four hours later, they were given an IV infusion of 0.04 mg/kg REMI over 40 min [15]. Euthanasia was performed six hours after the LPS administration.

*REMI group* (*n* = 8): Rats received 0.5–1 ml of saline i.p. After four hours, an IV infusion of 0.04 mg/kg REMI was administered over 40 min. Euthanasia was performed six hours after treatment.

For euthanasia, anesthesia was induced using 90 mg/kg ketamine (Ketalar, Pfizer, Türkiye) and 10 mg/kg xylazine (Xylazinbio 2%, Bioveta, Czech Republic). Euthanasia was conducted via surgical exsanguination, with blood collected from the inferior vena cava following an abdominal incision.

Tissues from the right ovaries, fallopian tubes, and uterus were excised and preserved in 10% formalin for histological and immunohistochemical analysis of TNF-α, NF-κB, and iNOS. The left ovaries were stored at -80 °C.stored for genetic analyses of Cas-3, Cas-9, Cas-12, NRF-2, and HO-1.

### Histopathological Examinations

The genital system—including the ovaries, fallopian tubes, and uterus —was thoroughly examined during necropsy. Tissue samples were collected and fixed in neutral buffered formalin. Routine tissue processing was carried out using a fully automated Leica ASP300S tissue processor (Leica Microsystems, Wetzlar, Germany). After embedding in paraffin wax, 5-µm thick sections were prepared using a Leica RM2155 rotary microtome (Leica Microsystems, Wetzlar, Germany) and stained with hematoxylin and eosin (HE). Histological evaluations were performed by a professional histopathologist from an external university, blinded to the experimental groups.

Histopathogical lesions of the ovaries, fallopian tubes, and were assessed using a semiquantitative grading system. Parameters evaluated included hyperaemia, edema, hemorrhage, inflammatory cell infiltration, degeneration, and epithelial loss. Each lesion was scored on a scale from 0 to 3 based on severity [8, 21, 22].

### Immunohistochemical Examinations

Immunohistochemistry was performed using the streptavidin-biotin complex method to evaluated iNOS [Anti-iNOS antibody [RM1017] (ab283655)], NF-κB [Anti-NF-κB p65 antibody (ab16502)], and TNF-α [Anti-TNF alpha antibody [RM1005] (ab307164)] expression in selected genital tract tissues. All primary and secondary antibodies were obtained from Abcam (Cambridge, UK), with primary antibodies diluted 1:100. Following a 60-minute incubation with primary antibodies, biotinylated secondary antibodies and a streptavidin–alkaline phosphatase complex were applied. The secondary antibody micro-polymer (ab236466) was used in conjuction with the Mouse and Rabbit Specific HRP/DAB IHC Detection Kit. Diaminobenzidine (DAB) served as the chromogen to visualize antigen expression.

Positive controls were included for each marker, and negative controls were prepared by omitting the primary antibody step. All evaluations were performed in a blinded manner. At ×40 magnification, 100 cells per group were counted to determine the percentage of positively stained cells for each marker. Image analysis was conducted using ImageJ software (version 1.48; National Institutes of Health, Bethesda, MD) was used to analyse the images, and statistical analysis was applied to the results. Morphometric measurements were performed using the Database Manual CellSens Life Science Imaging Software (Olympus Co., Tokyo, Japan).

### Reverse Transcription-Polymerase Chain Reaction (RT-qPCR)

RNA was isolated from homogenized tissues using the GeneAll RiboEx (TM) RNA Isolation Kit (GeneAll Biotechnology, Seoul, Korea), following the manufacturer’s instructions. The concentration and purity of RNA were measured using the BioSpec-nano nanodrop (Shimadzu Ltd., Kyoto, Japan). One microgram of total RNA was used for complementary DNA (cDNA) synthesis, performed with the A.B.T. ™ cDNA Synthesis Kit (Atlas Biotechnology, Türkiye) according to the kit protocol, using a thermal cycler. Primers were designed by identifying specific mRNA sequences and validating them through the NCBI database. The primer sequences used are listed in Table 1. Quantitative gene expression analysis was performed using a 2X SYBR Green Master Mix (Nepenthe, Türkiye) on a Bio-Rad CFX96 real-time PCR system (California, USA). GAPDH was used as the housekeeping gene.

**Table 1:** Gene accession numbers, product sizes, and primer sequences

Genes	Primary sequence	Product size	Accession number
GAPDH (HouseKeeping)	F: AGTGCCAGCCTCGTCTCATA	248 bp	NM_017008.4
	R: GATGGTGATGGGTTTCCCGT		
Caspase 3	F: GGCCGACTTCCTGTATGCTT	110 bp	XM_006253130.5
	R: CGTACAGTTTCAGCATGGCG		
Caspase 9	F: AGCCAGATGCTGTCCCATAC	148 bp	XM_039110693.1
	R: CAGGAACCGCTCTTCTTGTC		
Caspase 12	F: CTGCATCAGAATCCAGGGGA	212 bp	NM_130422.1
	R: TCGGCCTTCCTTCTCCATCA		
NRF2	F: GCCTTCCTCTGCTGCCATTAGTC	126 bp	NM_001399173.1
	R: TCATTGAACTCCACCGTGCCTTC		
HO-1	F: AGCCTGGTTCAAGATACTACCTC	240 bp	XM_039097470.1
	R: AGGCCCAAGAAAAGAGAGCC		

*F* Forward, *R* Reverse, *GAPDH* Glyceraldehyde-3-phosphate dehydrogenase, *Caspase 3* Cysteine-aspartic acid protease 3, *Caspase 9* Cysteine-aspartic acid protease 9, *Caspase 12* Cysteine-aspartic acid protease 12, *NRF2* Nuclear factor erythroid 2-related factor 2, *HO-1* Heme Oxygenase 1

Each reaction was prepared in a final volume of 20 µl, according to the manufacturer’s instructions, and analyzed in triplicate. The thermal cycling protocol included an initial denaturation at 94 °C for 10 min, followed by 40 cycles of denaturation at 95 °C for 15 s, and annealing/extension at 55 °C for 30 s. Relative mRNA expression levels were calculated using the 2^^−ΔΔCt^ method after normalization to GAPDH.

### Statistical Analysis

The Shapiro-Wilk test was initially applied to assess the normality of data distribution. As the data were normally distributed (*p* > 0.05), ANOVA was used to compare the groups. Variables are presented as mean ± standard deviation. Differences between groups in histopathological and immunohistochemical scores were analyzed using a one-way ANOVA followed by Tukey’s post hoc test. Statistical analyses were performed using Graphpad Prism version 8.0 (GraphPad Software, Inc., USA). A p-value of < 0.05 was considered statistically significant.

## Results

### Histopathological Evaluation Results

The LPS group exhibited mild to moderate hyperemia, while the CONT, REMI, and LPS-REMI groups appeared normal upon gross examination. The pathological abnormalities observed in the LPS group were attributable to the effects of LPS and were absent in the CONT group.

**Fig. 1:**
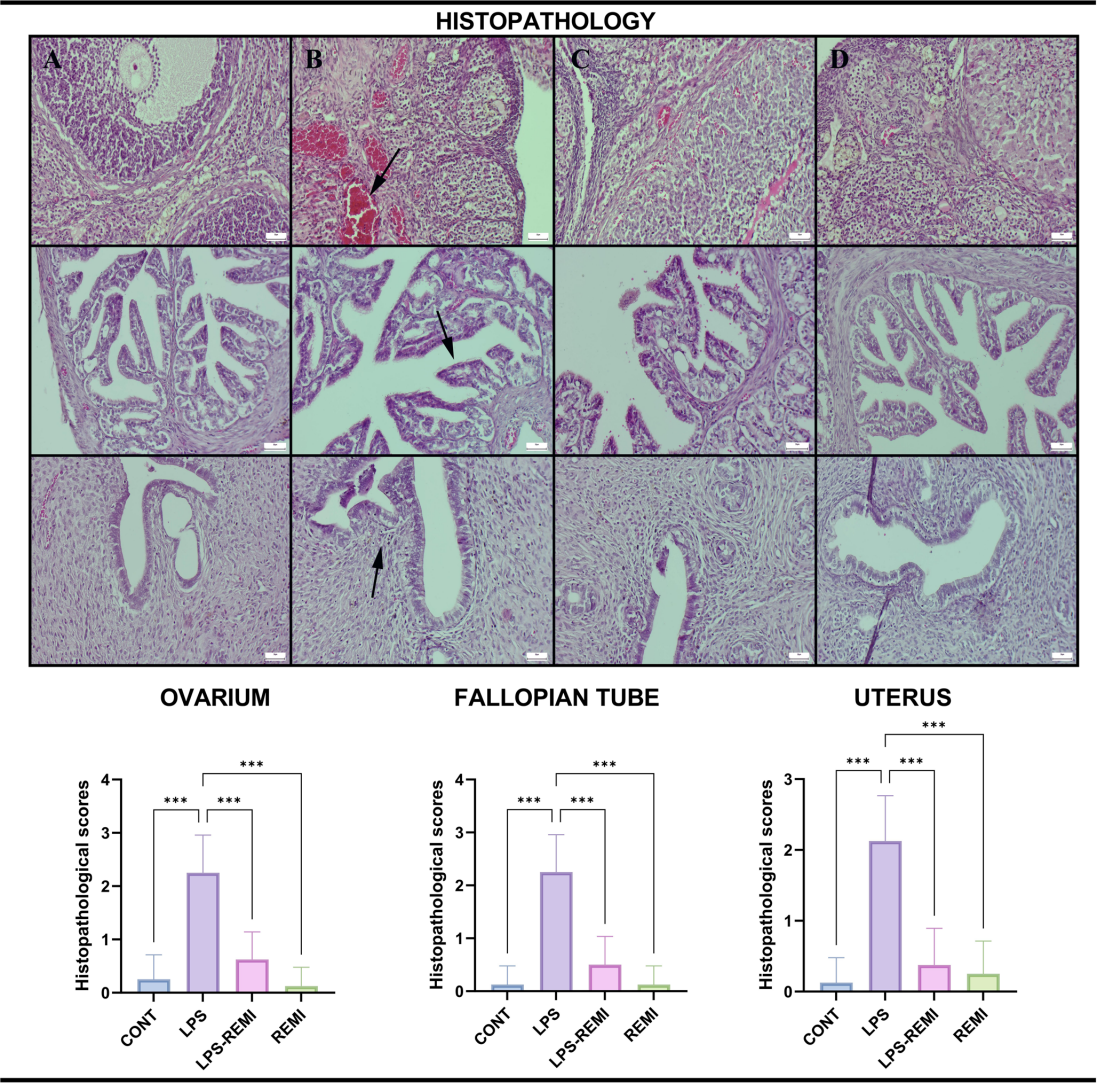
Histological analysis of the ovary (top row), fallopian tubes (middle row), and uterus (bottom row) across different groups. (**A**) The CONT group exhibited normal histology in all examined tissues. (**B**) The LPS group exhibited significant pathological alterations, including pronounced hyperemia in the ovary (arrow), cilia loss in the fallopian tube (arrow), and epithelial loss with inflammatory cell infiltration (arrow) in the endometrium. (**C**) The LPS-REMI group demonstrated fewer abnormalities, suggesting a protective effect of REMI. (**D**) The REMI group maintained normal tissue architecture. HE, scale bars = 50 μm

For histological evaluations, only animals in the diestrus phase were included to ensure hormonal consistency across groups. This phase was confirmed for all animals during histopathological examination. No pathological alterations were observed in the genital tissues of the CONT and REMI groups. In contrast, the LPS group showed marked hyperemia, edema, neutrophil infiltration, and varying degree of hemorrhage (*p* = 0.001). Additional findings included endometrial distruption, infiltration of inflammatory cells in the uterus, and a prominent loss of cilia in the fallopian tubes, all indicative of severe tissue injury caused by LPS exposure. Notably, these pathological changes were absent in the LPS-REMI group, suggesting that REMI effectively mitigated LPS-induced damage and exerted a significant protective effect (*p* = 0.001) (Fig. 1).

### Immunohistochemical Findings

**Fig. 2:**
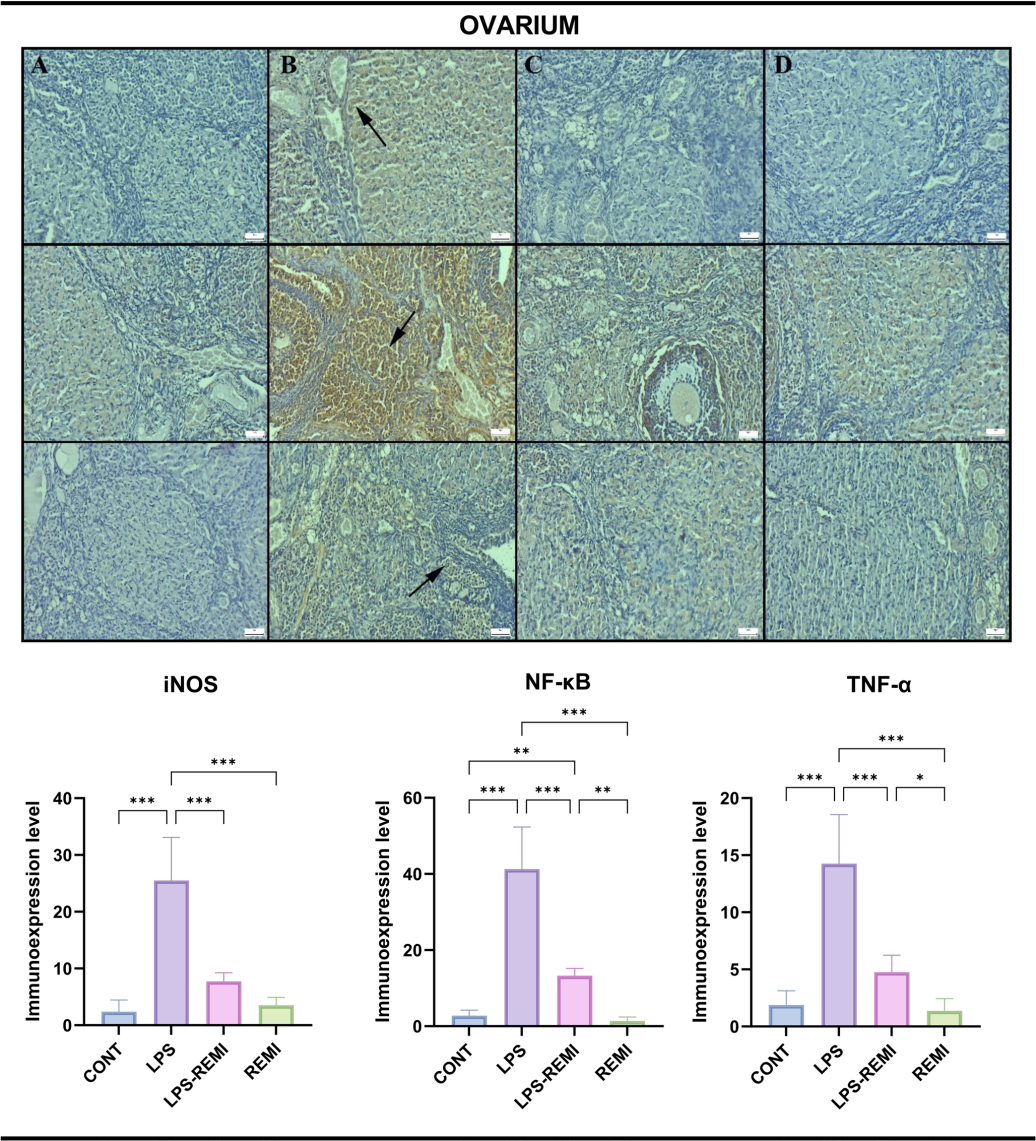
Immunohistochemical expression of iNOS (top row), NF-κB (middle row), and TNF-α (bottom row) in ovarian tissue. (**A**) Negative to very mild expression in the CONT group. (**B**) Significantly increased expression (arrows) in the LPS group. (**C**) Reduced expression in the LPS-REMI group. (**D**) Negative to very mild expression in the REMI group. Streptavidin-biotin peroxidase method. Scale bars = 50 μm. Data are presented as means ± standard deviation. Statistical analysis was performed using one-way ANOVA. *p ≤ 0.05, **p ≤ 0.01, ***p ≤ 0.001

**Fig. 3:**
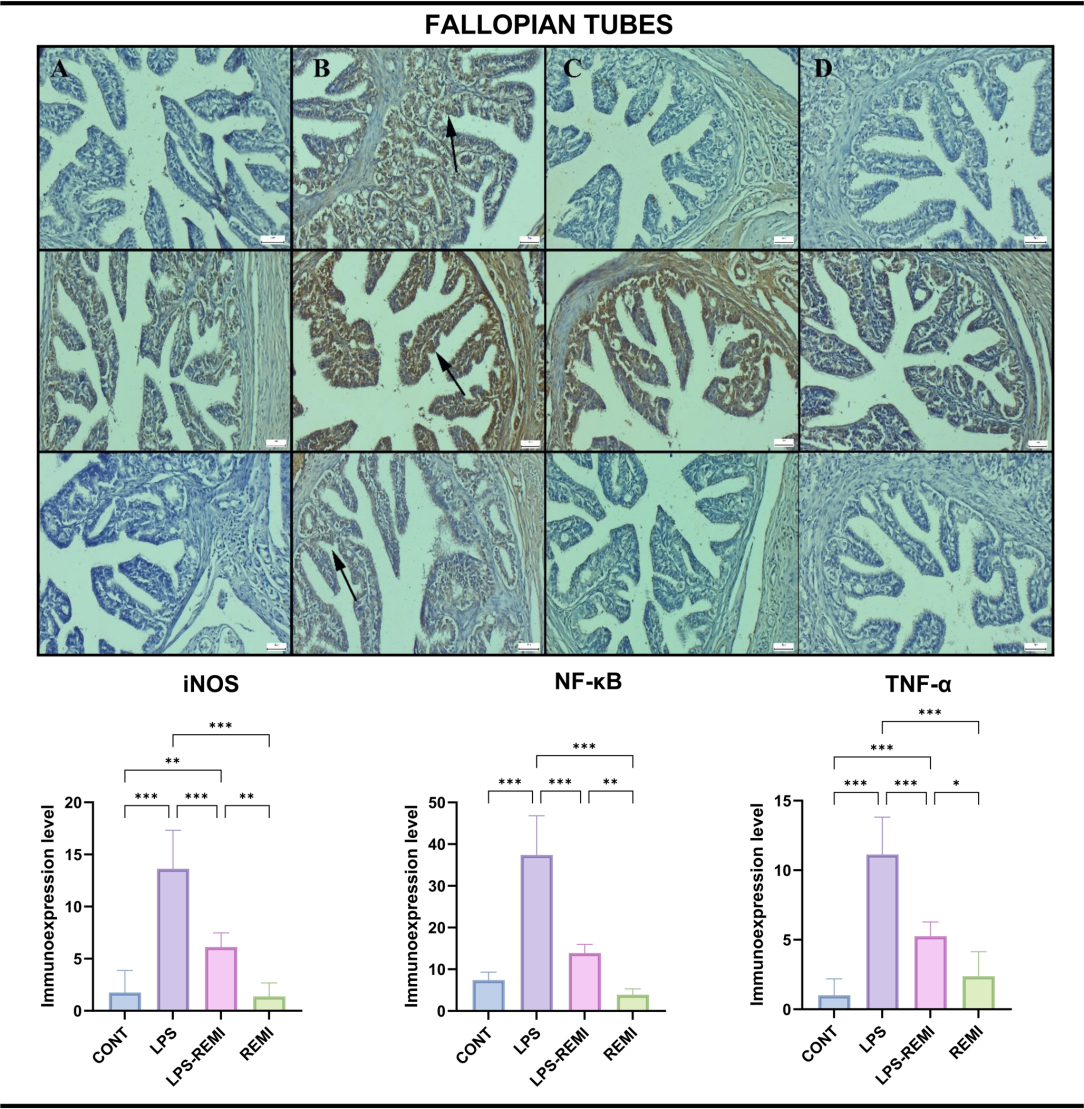
Immunohistochemical expression of iNOS (top row), NF-κB (middle row), and TNF-α (bottom row) in fallopian tube tissue. (**A**) Negative to very mild expression in the CONT group. (**B**) Significantly increased expression (arrows) in the LPS group. (**C**) Reduced expression in the LPS-REMI group. (**D**) Negative to very mild expression in the REMI group. Streptavidin-biotin peroxidase method. Scale bars = 50 μm. Data are presented as means ± standard deviation. Statistical analysis was performed using one-way ANOVA. *p ≤ 0.05, **p ≤ 0.01, ***p ≤ 0.001

**Fig. 4:**
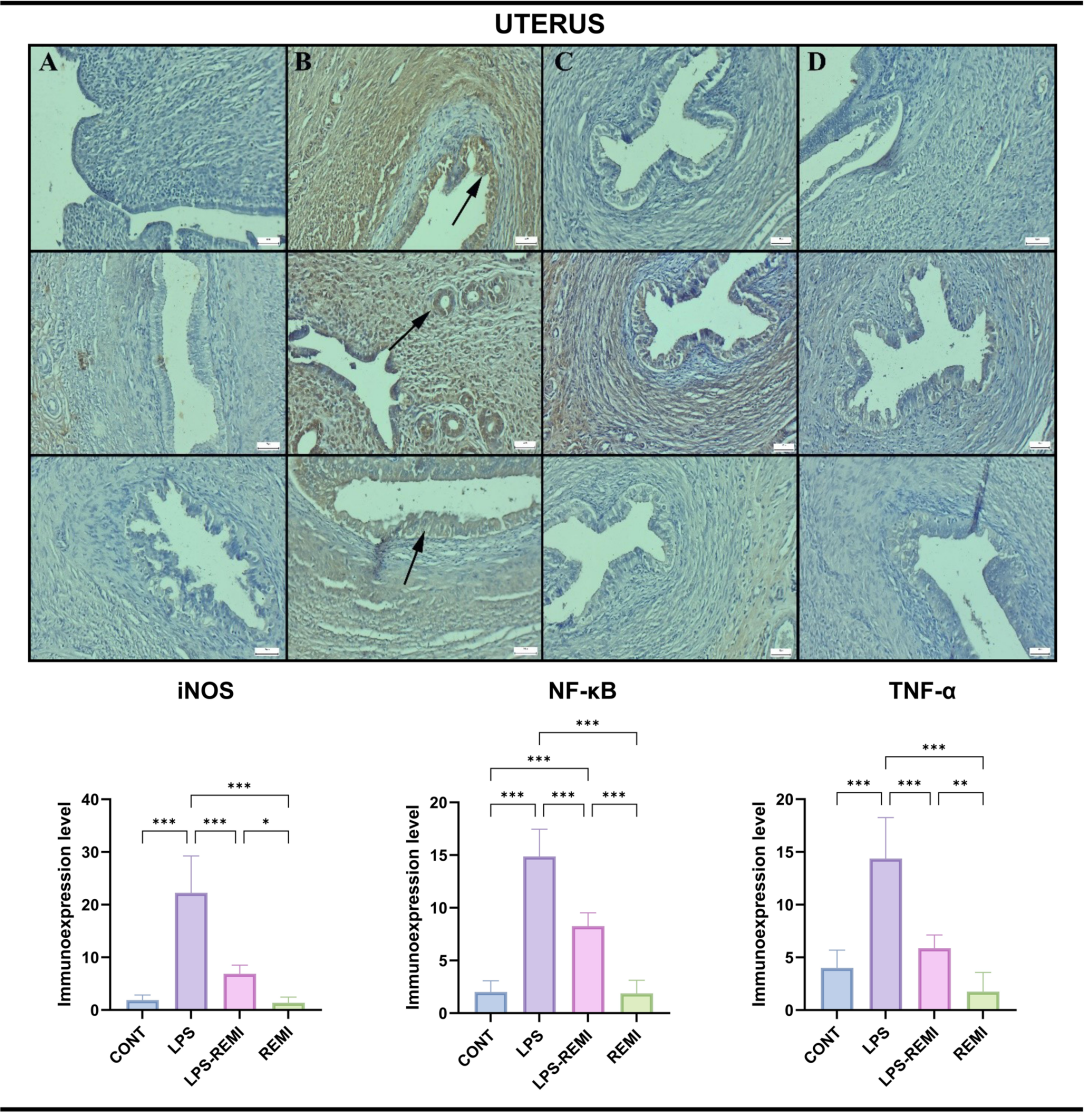
Immunohistochemical expression of iNOS (top row), NF-κB (middle row), and TNF-α (bottom row) in uterine tissue. (**A**) Negative to very mild expression in the CONT group. (**B**) Significantly increased expression (arrows) in the LPS group. (**C**) Reduced expression in the LPS-REMI group. (**D**) Negative to very mild expression in the REMI group. Streptavidin-biotin peroxidase method. Scale bars = 50 μm. Data are presented as means ± standard deviation. Statistical analysis was performed using one-way ANOVA. *p ≤ 0.05, **p ≤ 0.01, ***p ≤ 0.001

Immunohistochemical analysis revealed negative to modest expression levels of iNOS, NF-κB, and TNF-α in the uterus, fallopian tubes, and ovaries of the CONT and REMI groups. In contrast, the LPS group exhibited markedly elevated expression of all three markers, indicating a strong inflammatory response. REMI treatment significantly improved both immunohistochemical and histological outcomes, reducing tissue damage and inflammation. Figures 2, 3 and 4 provide a graphical representation of the statistical analysis of immunohistochemically positive cell counts.

All markers showed a statistically significant increase following LPS exposure, with the most pronounced expression observed in luteal and mesenchymal cells of ovarian tissue (*p* = 0.001). A similar expression pattern was observed in the uterus and fallopian tubes, where LPS treatment led to a significant elevation of TNF-α, NF-κB, and iNOS (*p* = 0.001). REMI treatment effectively reduced the expression of all markers, indicating its potential anti-inflammatory properties (*p* = 0.001).

Both mesenchymal and epithelial cells exhibited positive staining, with all markers localized exclusively in the cytoplasm. Notably, the REMI and CONT groups showed little to no expression of inflammatory markers, with no statistically significant difference between them. These findings suggest that REMI plays a crucial role in reducing the inflammatory response induced by LPS causes in the female reproductive system.

### PCR Results

**Fig. 5:**
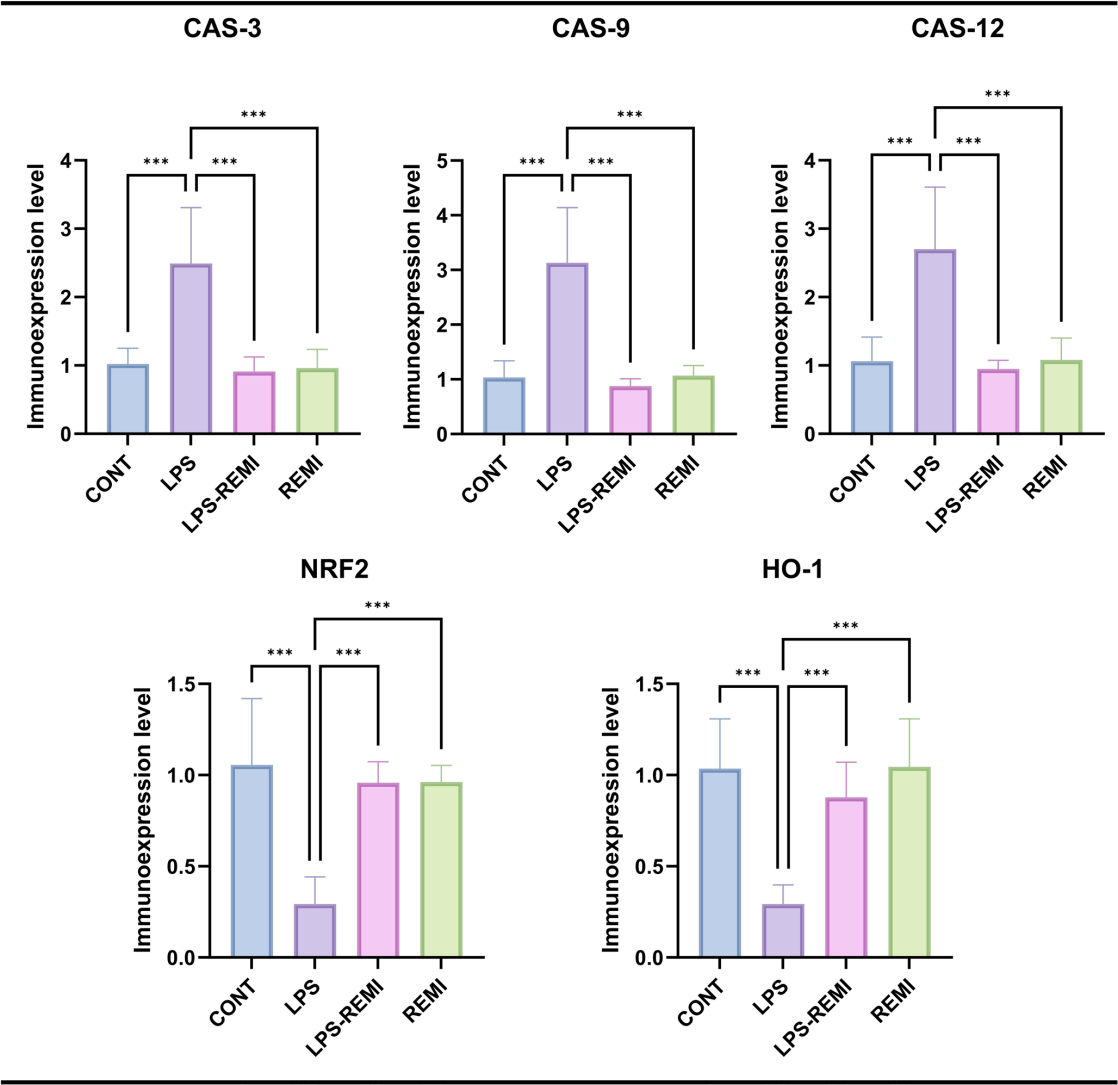
Expression levels of NRF2, HO-1, and caspases in genital tissues. *CAS-3* Caspase 3, *CAS-9* Caspase 9, *CAS-12* Caspase 12, *NRF2* Nuclear factor erythroid 2-related factor 2, *HO-1* Heme Oxygenase 1. Values are presented as means ± standard deviation. Statistical analysis was performed using one-way ANOVA. *p ≤ 0.05, **p ≤ 0.01, ***p ≤ 0.001

As indicators of inflammation and cellular damage, Cas-3, Cas-9, and Cas-12 immunoexpression levels were significantly higher in the LPS group compared to the CONT group (*p* = 0.001). In contrast, the LPS + REMI group showed significantly lower levels of Cas-3, Cas-9, and Cas-12 compared the LPS group (*p* = 0.001). As shown in Fig. 5, there was shows no discernible difference between the REMI and CONT groups.

NRF2, a key component of the antioxidant defence system, exhibited significantly reduced immunoexpression in the LPS group relative to the CONT group (*p* = 0.001). However, NRF2 levels were significantly increased in the LPS + REMI group compared to the LPS group (*p* = 0.001). No notable differences were observed between the CONT and REMI groups.

Similarly, HO-1 immunoexpression levels were significantly lower in the LPS group compared to the CONT group (*p* = 0.001). In contrast, HO-1 levels were significantly higher in the LPS + REMI group than in the LPS group (*p* = 0.001). There were no significant differences between the CONT and REMI groups.

## Discussion

This study investigated the effects of LPS-induced inflammation on the female reproductive system, specifically targeting the ovaries, fallopian tubes, and uterus. The results revealed that LPS exposure caused significant histopathological changes, including hyperemia, edema, inflammatory cell infiltration, and epithelial loss. These pathological alterations are consistent with pelvic inflammatory disease and endometrial injury, and they can lead to hormonal imbalances, impaired follicular development, and distrupted oocyte maturation, ultimately contributing to infertility. These findings are in line with previous reports indicating that LPS induces inflammatory responses in reproductive tissues and negatively affects folliculogenesis and ovulation [23]. Understanding these mechanisms underscores the importance of controlling inflammation to maintain reproductive health. Notably, treatment with REMI significantly ameliorated the observed histopathological alterations. The reduction in hyperemia, edema, and inflammatory infiltration, suggest that REMI exerts a modulatory effect on the inflammatory microenvironment of reproductive tissues. This is particularly relevant, as inflammation in ovarian and uterine tissues has been associated with reproductive disorders such as infertility, recurrent pregnancy loss, and suppression of the hypothalamic-pituitary-gonadal (HPG) axis [24].

Immunohistochemical analyses showed increased expression of TNF-α, NF-κB, and iNOS in the LPS group. TNF-α is a central pro-inflammatory cytokine that initiates and amplifies the inflammatory response, while iNOS—regulated by NF-κB—catalyzes the production of NO, contributing to oxidative stress and increased vascular permeability [2]. The upregulation of these markers confirms the activation of an inflammatory cascade in reproductive tissues following LPS exposure. In contrast, REMI administration significantly decreased the expression levels of TNF-α, NF-κB, and iNOS, supporting previous studies that report REMI’s anti-inflammatory effects via inhibition of NF-κB signaling pathways [14].

Inhibiting NF-κB activity is crucial in addressing inflammatory reproductive disorders, such as polycystic ovary syndrome (PCOS), recurrent pregnancy loss, and infertility. Chronic NF-κB activation has been implicated in the pathogenesis of these conditions, prompting research into NF-κB-targeted therapeutic strategies [2, 25]. Moreover, since NF-κB also plays a role in cervical dilation and uterine contractions, its premature activation may contribute to preterm birth, posing risks to both maternal and fetal health [26].

The observed histological improvements in the REMI group may also be attributed to reduced NO production, as evidenced by decreased iNOS expression. Inflammation is often accompanied by oxidative stress, which results from an imbalance between reactive oxygen species (ROS) production and antioxidant defense mechanisms. In this study, LPS exposure led to downregulation of HO-1 and NRF2—key components of the Nrf2/HO-1 pathway, which regulates oxidative stress and inflammatory responses [27]. Suppression of these markers indicates that LPS not only triggers oxidative stress but also compromises cellular antioxidant defenses, facilitating DNA damage, protein oxidation, and lipid peroxidation—factors that collectively impair reproductive function.

REMI treatment restored NRF2 and HO-1 expression levels, suggesting its capacity to enhance antioxidant responses. This is consistent with findings that REMI reduces oxidative stress and inflammation [11, 28]. Through upregulation of NRF2, REMI likely promotes the expression of antioxidant genes involved in glutathione synthesis and detoxification pathways, thereby protecting cells against oxidative injury.

Beyond oxidative damage, LPS-induced inflammation disrupts mitochondrial homeostasis and activates apoptotic pathways. Caspases —particularly Cas-3, Cas-9, and Cas-12. Cas-9 —play central roles in executing apoptosis. Cas-9 is involved in the intrinsic mitochondrial apoptotic pathway, while Cas-12 is activated under endoplasmic reticulum (ER) stress conditions [6, 7]. Mitochondrial dysfunction, a hallmark of LPS toxicity, results from ROS-mediated damage to mitochondrial membranes, leading to cytochrome c release and Cas-9 activation. Simultaneously, ER stress can trigger Cas-12 mediated apoptosis. The increased expression of these caspases in the LPS group confirms activation of both pathways. REMI administration significantly downregulated these markers, indicating its protective role against apoptosis likely by alleviating ER stress and modulating apoptotic signaling pathways [13, 29, 30].

REMI’s ability to attenuate oxidative damage, inhibit NF-κB, and suppress apoptosis highlights its broader role in preserving cellular homeostasis during inflammation. Preventing excessive apoptosis in reproductive tissues is essential for maintaining tissue structure, ovarian reserve, endometrial receptivity, and overall fertility potential.

Considering the findings of this study, the decreased expression of iNOS, NF-κB, and TNF-α in the LPS + REMI group was consistent with histopathological improvements, such as reduced inflammatory infiltration, edema, and tissue damage. This correlation supports the anti-inflammatory and tissue-protective effects of REMI in LPS-induced reproductive injury. Furthermore, PCR analyses revealed that REMI treatment significantly increased the expression of NRF2 and HO-1. As key regulators of the antioxidant response, activation of these factors contributes to the neutralization of ROS, suppression of pro-inflammatory mediators, and reduction of oxidative stress. These molecular findings aling with the observed histological improvements.

The results of this study also demonstrate that REMI, unlike many conventional anti-inflammatory agents, does not act on a single pathway but exerts a multifaceted mechanism by targeting inflammatory, oxidative, and apoptotic processes simultaneously. This broad-spectrum activity suggests that REMI may offer more effective protection in complex inflammatory conditions such as LPS-induced reproductive system injury.

In conclusion, LPS-induced inflammation in the female reproductive system results in notable histopathological damage, upregulation of pro-inflammatory and pro-apoptotic markers, and oxidative stress through ER stress pathways. REMI treatment effectively mitigates these effects, indicating its potential therapeutic utility in protecting reproductive health under inflammatory conditions. These findings support further investigations into REMI as a candidate for preventing inflammation-related reproductive dysfunctions and assessing its long-term efficacy and clinical relevance.

## Electronic Supplementary Material

Below is the link to the electronic supplementary material.

Supplementary Material 1

## Data Availability

The authors confirm that the data and materials supporting the findings of this study are available in the article.

## Declarations

### Animal Research

The study was designed and conducted according to ethical norms approved by the local animal experiments ethics committee of Suleyman Demirel University (Isparta, Türkiye). The Suleyman Demirel University local animal experimentation ethics committee gave its approval to the experimental protocol (No. 06.03.2025/03-450).
