# Supplementary figures and images for: Remifentanil Attenuates LPS-Induced Genital Tract Injury by Modulating Inflammation, Oxidative Stress, and Mitochondrial Gene Expression in a Rat Sepsis Model
Source: Reprod Sci. 2025 Jul 15;32(8):2583–94. doi: 10.1007/s43032-025-01930-7 (PMC12360976; doi:10.1007/s43032-025-01930-7)

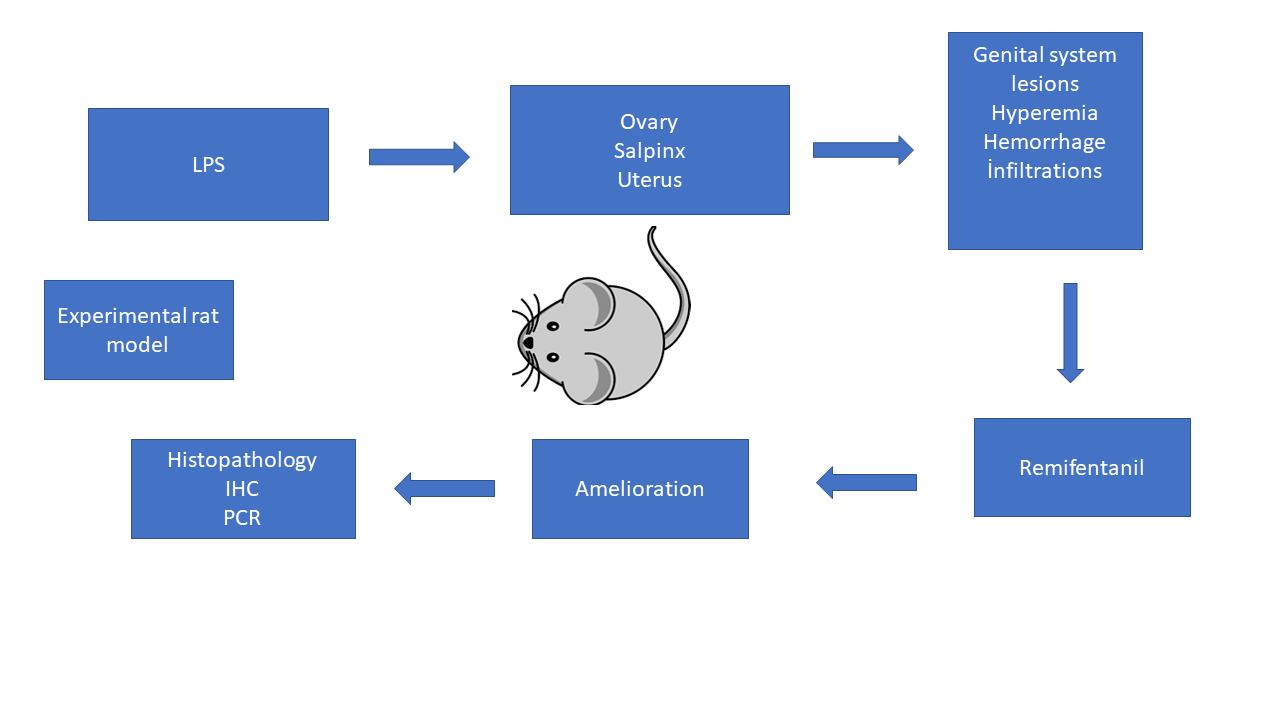

Supplement: Supplementary file 1 — Supplementary Material 1 [file 43032_2025_1930_MOESM1_ESM.tif]
